# A national cross-sectional study of adherence to timely mammography use in Malta

**DOI:** 10.1186/s12885-018-4278-9

**Published:** 2018-03-27

**Authors:** Danika Marmarà, Vincent Marmarà, Gill Hubbard

**Affiliations:** 1 Faculty of Health Sciences, University of Stirling, Room E9, Pathfoot, Stirling, FK9 4LA Scotland; 2 Ministry for Health, Cancer Care Pathways Directorate, Sir Anthony Mamo Oncology Centre, Level -1, Dun Karm Psaila Street, Msida, MSD 2090 Malta; 3 Faculty of Economics, Management and Accountancy, University of Malta, Room 408, Msida, MSD 2080 Malta

**Keywords:** Mammography, Attendance, Adherence, Recent, Health beliefs, Illness perceptions

## Abstract

**Background:**

Routine mammography improves survival. To achieve health benefits, women must attend breast screening regularly at recommended time intervals. Maltese women are routinely invited to undergo mammography at three-year intervals at an organized breast screening programme (MBSP) or can opt to attend a private clinic. Previous research shows that health beliefs, particularly perceived barriers, were the most significant predictors of uptake to the first MBSP invitation. Whether these beliefs and other factors are predictive of adherence with recommended time intervals for mammography at organized or private screening in Malta is unknown. For the first time, this paper explores the predictors for Maltese women screened within or exceeding the recommended three-year frequency in organized or private screening in Malta.

**Methods:**

Information was obtained from a cross-sectional survey of 404 women, aged 50 to 60 years at the time of their first MBSP invitation, where women’s characteristics, knowledge, health beliefs and illness perceptions were compared. The main variable of interest was women’s mammography attendance within a three-year interval (ADHERENT) or exceeding three years (NON-ADHERENT). Data were analysed using descriptive statistics, chi-square test, Mann Whitney test, Independent Samples t-test and Shapiro Wilk test.

**Results:**

At the time of the survey, 80.2% (*n* = 324) had been screened within three years (ADHERENT), 5.9% (*n* = 24) had exceeded the three-year frequency (NON-ADHERENT) while 13.9% (*n* = 56) never had a mammogram. No significant associations were found between ADHERENT or NON-ADHERENT women in relation to sociodemographic or health status variables (*p >* 0.05). Knowledge of screening frequency was significantly associated with women’s mammography adherence (χ2 = 5.5, *p* = 0.020). Health beliefs were the strongest significant predictors to describe the variance between ADHERENT and NON-ADHERENT screeners. When Mann Whitney test and Independent Samples t-test were applied on mammography adherence, perceived barriers and cues to action were found to be the most important predictors (*p* = 0.000, *p* = 0.039 respectively).

**Conclusions:**

To increase routine and timely mammography practices, women who are non-adherent to recommended time frequency guidelines should be targeted, together with their health beliefs, predominantly perceived barriers and cues to action.

**Electronic supplementary material:**

The online version of this article (10.1186/s12885-018-4278-9) contains supplementary material, which is available to authorized users.

## Background

Breast cancer (BC) is the most common cancer among women worldwide [1, 2]. The World Health Organization (WHO) has identified prevention, early detection and managing the cancer trajectory as the three pillars in the reduction and control of the global cancer burden [3]. Based on global evidence from randomized controlled trials [4, 5], early detection through mammography screening has been documented to significantly decrease BC mortality rates [6–8], and can lead to early treatment and reduce its negative side-effects [9].

In Malta, BC is the most common type of cancer among women. Around 280 cases have been diagnosed each year in the last decade [10] with the Maltese nation ranking 18th place with the highest incidence of BC in 2012 (85.9 per 100,000) [11]. The Maltese Breast Screening Programme (MBSP) was established in 2009 for women aged 50–60 years every three years [12] and has now expanded its age range to include women aged 61–67 years. Prior to the MBSP, women in Malta could use private mammography (there are currently 7 private practices offering mammography in Malta).

Despite the availability of the MBSP, a number of women still do not attend for mammography at the MBSP or may not attend at recommended intervals. This is evidenced by data from our national cross-sectional survey [13] showing that the uptake rate for first round BS at the MBSP was lower than the European target rate of 70% [9], and similarly for re-attendance, evidenced in our pilot study on the second BS round in Malta [14]. These programmes can only be effective and indeed cost-effective [15] if the attendance of the target screening population is consistent with recommended intervals [16–18] in order to achieve health benefits [8, 16, 19].

Currently, organized breast screening (BS) programmes are offered for free to asymptomatic women by many countries in Western Europe and North America [20], with time intervals between mammograms depending on the varying recommendations of various countries [21]. In Europe, the EU Council recommends a two-year interval to women aged 50–69 years [9, 22]. However, countries implement these recommendations as they consider fit [20]. For instance, Norway adheres to the recommended EU thresholds, while a biennial nationwide screening programme for women aged 50–75 is offered in the Netherlands [23] and regionally organized screening programmes are offered in Switzerland for women over 50, with the age limit varying between 69 and 74 years [24]. Notable exception for the screening interval is by United Kingdom and Malta who opted for a three-yearly screening frequency [25].

Substantial disparity remains to date across countries on attendance at regular time intervals [2] with recent and regular attendance being studied less often than initial attendance [17, 26, 27]. For instance, the more privatized system in the United States may enable less access to mammography than the social health care system found in the United Kingdom [28], suggesting that national context is important and worth exploring. The Maltese National Health System (NHS) adopts a mixed model approach comprising elements from both the public (organized) and private sectors and this is one possible reason for non-participation in the organized screening programme (MBSP) or non-attendance at recommended intervals. Prior to the MBSP rollout across Malta, asymptomatic women could self-refer privately for mammography and symptomatic women were referred by a general practitioner (GP), breast surgeon or gynaecologist either to the public symptomatic breast unit or to the private sector for mammography. Despite having the availability and efficiency of nationally-organised screening programmes, some women may still opt for the service privately and are considered asymptomatic attendees to opportunistic screening [29, 30] but are non-compliant in the context of invitation-based BS [27]. Similarly, screening mammograms taken and read in private clinics [30] remain widely used in America and in European countries such as France, Luxembourg and Switzerland [30–33].

To date, we are not aware of any study that has explored attendance to mammography screening according to recommended time-intervals at organized or private practices. Therefore, in order to understand if Maltese women are adherent with recommendations for BS, we analysed primary survey data in an effort to describe the adherence rates. Hence, in order to analyse the differences between timely mammography adherence and non-adherence to current time interval recommendations (three-year interval), we explored several determinants, mainly health beliefs and illness perceptions. We built on the findings of our prior study [13] which suggests that health beliefs and illness perceptions vary between women who accept or refuse a BS invitation to the organized programme. ‘Strengthening the Reporting of Observational Studies in Epidemiology’ (STROBE) guidelines [34] [see Additional file 1], have guided the study findings in this article. This study will help to inform public health experts, policy-makers and screening management to tackle regulated routine attendance in their population-based screening programmes.

### Theoretical framework

#### Health beliefs

Health behaviour takes place when a threat is recognized as a result of a health problem [35] and is manipulated by the individual’s perception of that threat [36]. The Health Belief Model (HBM) has often been recommended when dealing with behaviours that evoke illness, such as BC [37], and is thus an excellent fit for addressing the health beliefs and perceptions of BS among women. The HBM consists of the following six main variables: perceived susceptibility, perceived severity, perceived benefits, perceived barriers, cue to action, and self-efficacy [38]. Individuals will take action to prevent, to screen for, or to control BC if they perceive themselves to be susceptible to the condition, if they believe in the seriousness of the potential consequences, if they believe the course of action would reduce their susceptibility to or the severity of the condition, and if they believe that the anticipated benefits to taking the action outweigh the barriers [38]. Based on the HBM, engaging in mammography will be predicted by women’s perceptions about BC derived from their knowledge about the disease [39]. Thus, it is significant for healthcare providers to increase knowledge through education about BC and the importance and benefits of BS such as early detection, reduced mortality and improved survival.

Several researchers have used standardized measures of HBM constructs, such as Champion’s HBM scales for mammography screening (CHBMS-MS) [35, 40, 41] in order to determine the relationships between health beliefs and health behaviours. These scales have been translated and tested for reliability and validity in diverse populations such as Iranian [40], Lithuanian [42], Malaysian [43], Arabic [44], Korean [45], Chinese-Australian [46], African-American [47], Spanish-speaking American women [48] and Spanish women [41]. However, the variation in BS behaviours is limitedly explained through HBM, since the impact of emotions (such as fear) [49] is not considered, nor does it accommodate social and environmental influences of past behaviour [50]. This is why other models, such as the Common-Sense Model (CSM) of self-regulation, have been utilised to understand BS uptake [51] and to explain the variations in physical and psychological adjustment to BC and disease outcomes [52, 53].

#### Illness perceptions

According to the Common-Sense Model (CSM)*,* illness perceptions are related to the cognitive (i.e. beliefs, thoughts, ideas) and emotional (i.e. feelings) representations derived from the experience of an illness or illness-related symptoms [54]. Each individual is known to have his/her own beliefs about health / illness due to similar but unique experiences [55]. Hence, an individual’s behaviour can be affected by the assessment of symptoms and knowledge, beliefs and risk perceptions [56]. In regard to healthy people, illness perceptions can serve as guides for behaviour in relation to prevention [57] and appear to be precursors of screening behaviour [56].

The utility of the CSM has been extensively investigated quantitatively following the development of a questionnaire, the Illness Perception Questionnaire (IPQ) [58], which addresses the following five key dimensions: symptoms and names (identity), severity of pain and impact on life functions (consequences), expected duration or expected age of onset (timeline), whether the disease was perceived as preventable, curable, or controllable (control/cure) and infection or genetics (internal and external causes), in Leventhal’s self-regulatory model [59]. Following advancement in theory and measurement of the constructs related to the CSM, the IPQ has been revised, expanded and renamed as IPQ-R [60] with the inclusion of new dimensions, such as the illness coherence scale in order to better evaluate the overall meaning of the illness for the patient. In addition, the content of the original cure/control component from the IPQ was treated separately in the IPQ-R as the ‘personal control’ scale i.e. about personal abilities to control the illness and ‘treatment control’ scale i.e. the efficacy of treatment to cure or manage the illness [61]. Also, the timeline dimension was differentiated into two: (a) timeline (acute/chronic) i.e. beliefs about the relative chronicity of the illness and (b) timeline (cyclical) i.e. beliefs about the fluctuation in symptoms and temporal illness changeability [57]. An important inclusion in IPQ-R was the measure of emotional representations (related to the cognitive components of illness representations) [57, 60].

## Methods

The full details of the methods are described in detail elsewhere [13]. In brief, a cross-sectional survey was undertaken in Malta in 2015 and data were drawn retrospectively from a nationally representative sample of eligible women (*n* = 404 with 95% confidence level and a 5% significance level), aged 50–60 years at the time of their first invitation at the MBSP with no personal history of BC. From our total sample, 60% were attendees (*n* = 243) to breast screening and 40% were non-attendees (*n* = 161) to the first call. This is an actual representation of the uptake to the first screening invitation. From every sub-population, the sample was selected by stratified random sampling, i.e. stratified based on district and age to give a true representation based on the demographics of attendance and non-attendance to the first screening round. Hence, all individuals were selected at random based on the above percentages and stratification.

Screening mammography uptake in the past three years was self-reported for women who opted to go privately but for those who had attended the MBSP, attendance or non-attendance was verified through screening records. Participation was voluntary and verbal informed consent was obtained by telephone by a research assistant. Full recruitment details are described in our prior paper [13]. In order to carry out the study according to our methods and consent procedure, formal ethical approval was sought and obtained from the School Research Ethics Committee at the University of Stirling (SREC14/15-Paper No.18v4) and from the Maltese Health Ethics Committee (HEC 02/2015).

### Measures

Based on previously validated questionnaires (CHBMS-MS and IPQ-R) [35, 60], our study instrument, a 121-item questionnaire was initially translated from English into the Maltese language, adapted and pilot-tested among Maltese women [62] after securing written permission from the respective authors. A full description of the measures has been published in a previous article [13].

### Classification of variables

Women were asked with a yes/no response if they had a mammogram within the past three years (ADHERENT) or whether they had exceeded the three-year frequency (NON-ADHERENT). Furthermore, they were asked to identify the location of their mammogram if they had undergone the screening test recently.

### Statistical analysis

Throughout the analyses, basic statistics were presented through the use of mean values or percentages. A Chi-square test was used to test for any significant associations between two categorical variables. The Shapiro-Wilk test was applied on all 14 constructs to determine whether these variables are normally distributed. Since only the variable (Causes of BC) was normally distributed, the Independent Samples t-test (parametic test) was used for the latter construct to compare two independent samples, while the Mann-Whitney test was used for the non-normal distributed variables (non-parametric test) i.e. for all the other 13 constructs which were not normally distributed (*p* < 0.001). Missing data was minimal and reported in our previous paper [13]. Statistical significance was established at *p* < 0.05 for all analyses.

## Results

### Sample characteristics

The mean age was 54.6 years ±2.8 years (SD). A table presenting the sample characteristics for the total sample (*n* = 404) is available in our previous published paper [13]).

### Mammography screening practices

**Fig. 1:**
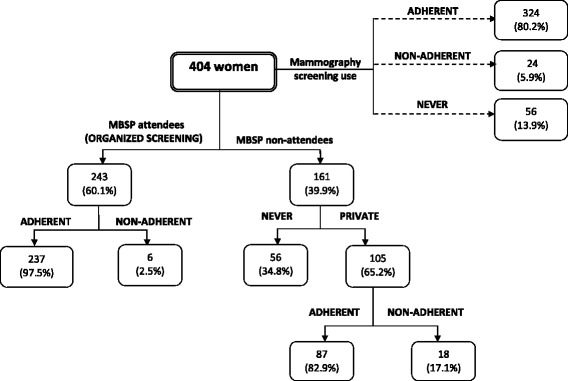
Mammography use in Malta

Figure 1 presents the mammography screening practices by Maltese women. From the total sample of 404 women, 80.2% (*n* = 324) had a recent mammogram (ADHERENT), 5.9% (*n* = 24) had exceeded the three-year frequency (NON-ADHERENT) and 13.9% (*n* = 56) never had a mammogram.

Out of the 404 women, 60.1% (*n* = 243) attended the MBSP and 39.9% (*n* = 161) did not. Out of 39.9% (*n* = 161) of women who did not undergo a mammogram at the MBSP, there were 65.2% (*n* = 105) who underwent mammography elsewhere (at a private practice), of which 82.9% (*n* = 87) had a mammogram within three years (ADHERENT) while 17.1% (*n* = 18) had a mammogram that exceeded the recommended regular three-year frequency (NON-ADHERENT).

Out of the 60.1% (*n* = 243) of women who underwent a mammogram at the MBSP, 97.5% (*n* = 237) had a recent screening mammography at the MBSP (ADHERENT), while 2.5% (*n* = 6) exceeded the three-year frequency (NON-ADHERENT). When applying a Chi-square test to compare NON-ADHERENCE to private practice versus NON-ADHERENCE to MBSP (17.1% versus 2.5%), this result was found to be significantly associated (χ2 = 24.6, *p* = 0.000).

Those who never attended for mammography anywhere (*n* = 56) were excluded from this analysis as this group was already analysed in further detail in our previous published paper on lifetime mammography use [63].

### ADHERENT versus NON-ADHERENT subgroup analyses

Chi-square tests were performed to explore associations between ADHERENT and NON-ADHERENT attenders and the following variables: sociodemographic factors, health status, knowledge, health beliefs and illness perceptions.

### Sociodemographic characteristics and health status

No significant associations were found between ADHERENT or NON-ADHERENT women in relation to sociodemographic or health status variables (*p >* 0.05).

### Knowledge of breast screening frequency

Knowledge of BS frequency was significantly associated with women’s adherence to mammography screening (χ2 = 5.5, *p* = 0.020). The main difference arises from those who said they were ‘unsure’ about the recommended frequency, where 12.5% of the non-adherent group were unsure about the recommended BS frequency while only 3.1% of the adherent group were unsure of the recommended time interval.

### Health beliefs

Some sub-scale items for perceived barriers and cues to action were found to be statistically significant (*p* < 0.05) when comparing adherent versus non-adherent women (Table 1). Non-adherent women were undecided on the following items when compared to adherent women: ‘having a routine mammogram would make you anxious about BC’ (*p* = 0.040), ‘if your GP advises you to attend, you will attend’ (*p* = 0.038), ‘hearing about BC and BS in the media or news makes you think about getting a mammogram’ (*p* = 0.030), or ‘reminder letters’, ‘reminder phone calls or text messages’ would help you to get a mammogram’ (*p* = 0.000 respectively). Women who fear or distrust the medical team (*p* = 0.003) or who feel they have too many other problems in life (*p* = 0.001) tend to attend less frequently. Women who do not agree that reminder letters, reminder phone calls or text messages would help them to get a mammogram (*p* = 0.000 respectively) also tend to attend less frequently to mammography.

**Table 1:** Health Belief items

	ADHERENT versus NON-ADHERENT	
Health Beliefs	χ2	*p*-value
There is no possibility of getting breast cancer *(r)*	5.5	0.239
Your chances of getting breast cancer are high	0.3	0.960
There may be the possibility of developing breast cancer in your lifetime	7.8	0.055
When you get a mammogram, you feel good about yourself	5.9	0.115
When you get a mammogram, you do not worry as much about breast cancer	3.6	0.302
Having a mammogram will help you find lumps early in your breasts	0.9	0.819
If you find a lump through a mammogram, the treatment for breast cancer may not be as bad	0.7	0.863
Having a mammogram will decrease your chances of dying from breast cancer	1.2	0.744
Having a mammogram will help you find a lump before it can be felt by yourself or a health professional	1.5	0.676
Having a routine mammogram would make you anxious about breast cancer	8.3	0.040*
Having a routine mammogram would make you worry	3.2	0.522
You fear having a mammogram because you might find out that something is wrong	5.3	0.257
You fear having a mammogram because you do not know the procedure or what to expect	2.8	0.418
You fear having a mammogram because you know someone (family or friend) with breast cancer	7.0	0.136
It is embarrassing for you to have a mammogram	6.0	0.055
Undergoing mammography will be painful or uncomfortable	3.8	0.284
Having a mammogram is time consuming	2.7	0.258
You are discontent with Breast Screening personnel as they have been rude to you	n/a	n/a
You have fear or distrust in the medical team	13.9	0.003*
Having a mammogram would expose you to unnecessary radiation	4.7	0.197
You have too many other problems in your life than to get a mammogram done	14.9	0.001*
You are not old enough to have a mammogram periodically	0.4	0.823
If your GP advises you to attend for a mammogram, you will attend	8.4	0.038*
If your relatives or friends advise you to attend for a mammogram, you will attend	1.3	0.741
If someone close to you has been diagnosed with breast cancer, you will attend for a mammogram	3.2	0.362
Hearing about breast cancer and breast screening in the media or news makes you think about getting a mammogram	8.9	0.030*
Reminder letters would help you to get a mammogram	20.9	0.000*
Reminder phone calls or text messages would help you to get a mammogram	20.9	0.000*
Routine educational talks regarding breast cancer awareness would help you to get a mammogram	6.7	0.820
You feel confident that if you had a mammogram done, any abnormalities in your breasts will be detected	4.7	0.318
You can arrange other things in your life to get a mammogram	1.5	0.821
In case you need a mammogram, you will find a place to get it done	1.8	0.752
You can make an appointment for a mammogram	1.7	0.800
You can arrange transportation to get a mammogram	1.6	0.812
You can talk to people at the breast screening centre about your concerns	n/a	n/a
You can find a way to pay for a mammogram if you need to	2.3	0.511

*(r)* = reverse scored

*Significant at α = 0.05

Chi-square test was applied for all health beliefs; hence the categorical answers were used to apply this test for association. For each question, respondents were asked to select a number between 1 and 5, where 1 = strongly disagree and 5 = strongly agree. For certain items, responses were re-grouped to ensure the feasibility of the Chi-square test

### Illness perceptions

Women who are undecided on the following subscale items attend less frequently for mammography: ‘your mental attitude’ (*p* = 0.008), ‘family problems or worries’ (*p* = 0.035), your emotional state’ (*p* = 0.000), ‘your personality’ (*p* = 0.006), and ‘you get anxious when you think about BC’ (*p* = 0.044) (Table 2). Mann Whitney test and Independent Samples t-test were applied to compare ‘ADHERENT’ and ‘NON-ADHERENT’ mammography use against all 14 constructs, showing a statistically significant difference in perceived barriers and cues to action (*p* = 0.000, *p* = 0.039 respectively) between adherent and non-adherent women (Table 3).

**Table 2:** Illness Perception items

	ADHERENT versus NON-ADHERENT	
Illness Perceptions	χ2	*p*-value
The presence of a lump or thickening in the breast	3.2	0.361
Nipple discharge	4.1	0.254
Sudden nipple retraction	7.0	0.072
Change in shape or appearance of the nipple	7.9	0.052
Breast swelling, dimpling, redness or soreness of the skin	3.6	0.305
Skin changes of the breast	4.7	0.193
A sudden change in breast size	1.5	0.682
Aching breasts	6.2	0.185
Stress or worry	2.8	0.250
Your mental attitude (e.g. thinking about life negatively)	12.0	0.008*
Family problems or worries	6.7	0.035*
Overwork	7.5	0.057
Your emotional state (e.g. feeling down, lonely, anxious, empty)	22.0	0.000*
Your personality	12.3	0.006*
Hereditary - it runs in the family	3.2	0.360
Diet or eating habits	1.9	0.590
Poor medical care in the past	1.4	0.699
Your own behaviour	3.8	0.282
Ageing	0.8	0.663
Smoking	0.5	0.927
Alcohol	0.0	0.979
A germ or virus	2.9	0.234
Pollution in the environment	2.8	0.428
Altered immunity	0.4	0.933
Chance or bad luck	1.0	0.908
Accident or injury	1.2	0.875
Breast cancer will last a short time	0.6	0.904
Breast cancer is likely to be permanent rather than temporary	4.8	0.089
A patient with breast cancer goes through cycles in which her illness gets better and worse	1.6	0.800
Breast cancer has major consequences on a patient’s life	2.1	0.559
Breast cancer will not have much effect on your life	2.4	0.662
Breast cancer would strongly affect the way others see you	4.4	0.351
Breast cancer has serious economic and financial consequences	0.8	0.840
Breast cancer would strongly affect the way you see yourself as a person	2.7	0.446
Breast cancer would threaten a relationship with your husband or partner	3.6	0.461
If you had breast cancer, your whole life would change	0.6	0.902
If you developed breast cancer, the chances of living a long life would decrease	0.8	0.844
There is a lot which you can do to control the symptoms if Breast Cancer occurs	1.3	0.869
The course of Breast Cancer will depend on your actions	1.7	0.646
Your actions will have an effect on the outcome of Breast Cancer	1.1	0.787
There is no treatment that will help to improve Breast Cancer	4.0	0.406
The treatment provided will be effective in controlling or curing Breast Cancer	0.5	0.926
The negative effects of Breast Cancer can be prevented or avoided by the treatment given	1.0	0.914
You have a clear picture and understanding of Breast Cancer	2.6	0.455
Breast Cancer is a mystery to you	1.7	0.786
You get anxious when you think about Breast Cancer	8.1	0.044*
Breast Cancer makes you feel afraid	0.7	0.875
You get worried when you think about Breast Cancer	0.7	0.871

*Significant at α = 0.05

Chi-square test was applied for all health beliefs; hence the categorical answers were used to apply this test for association. For each question, respondents were asked to select a number between 1to 5, where 1 = strongly disagree and 5 = strongly agree. For certain items, responses were re-grouped to ensure the feasibility of the Chi-square test

**Table 3:** Comparisons between the frequency of mammography use and health beliefs/illness perception constructs

	ADHERENT (*n* = 324)	NON-ADHERENT (*n* = 24)	Test Statistic	*p*-value
Perceived Susceptibility	M = 9.6, SD = 1.0	M = 9.4, SD = 0.9	3641.0^a^	0.577
Perceived Benefits	M = 24.0, SD = 1.8	M = 23.9, SD = 1.3	3515.0^a^	0.387
Perceived Barriers	M = 27.2, SD = 4.7	M = 31.1, SD = 5.0	5540.5^a^	0.000*
Cues to action	M = 27.4, SD = 3.2	M = 26.0, SD = 3.5	2919.0^a^	0.039*
Self-Efficacy	M = 24.8, SD = 2.7	M = 24.5, SD = 2.1	3666.5^a^	0.615
Breast Cancer Identity	M = 30.6, SD = 2.1	M = 30.3, SD = 3.4	4136.5^a^	0.582
Causes of Breast Cancer	M = 55.9, SD = 7.2	M = 57.0, SD = 6.9	-0.7^b^	0.467
Cancer Timeline: Acute/Chronic	M = 6.1, SD = 0.9	M = 5.9, SD = 1.0	3515.5^a^	0.402
Cancer Timeline: Cyclical	M = 3.6, SD = 0.7	M = 3.7, SD = 0.6	4271.0^a^	0.327
Consequences	M = 28.2, SD = 2.5	M = 28.5, SD = 2.7	4247.5^a^	0.446
Personal Control	M = 11.8, SD = 0.8	M = 11.9, SD = 0.6	3905.5^a^	0.951
Treatment Control	M = 9.9, SD = 0.7	M = 10.0, SD = 0.5	4166.5^a^	0.397
Illness Coherence	M = 6.9, SD = 1.2	M = 7.3, SD = 0.9	4538.0^a^	0.139
Emotional Representations	M = 12.2, SD = 2.1	M = 12.7, SD = 1.8	4348.0^a^	0.320

*Significant at α = 0.05

^a^Mann Whitney test

^b^Independent Samples t-test

The findings show that for women who were NON-ADHERENT to the three-year time frequency for mammography use perceive higher barriers and lower cues to action than ADHERENT women.

## Discussion

This study made possible an understanding of the determinants of timely BS behaviour in Malta which may aid the development of evidence-based and culturally-sensitive interventions for the Maltese population. Our findings show that women who have previously participated in BS practices may already understand the screening benefits for BC, have come to terms with barriers to undergo mammography, and have confidence in their abilities to get screened [2] and thus attend for mammography at the recommended time intervals. Similarly, findings by Moodi et al. [64] suggest that women who previously had at least one mammogram in their lifetime had higher levels of health motivation, perceived benefits, and perceived self-efficacy to mammography screening and fewer perceived barriers to having a mammogram. This further proves that previous mammography use strongly predicts subsequent screening [2, 14, 64].

Our study showed that there were some Maltese women who did not attend for mammography in a timely manner. In terms of (self-initiated) behaviour with mammography, this could be due to the fact that not all women may view this as a positive action to improving health outcomes. Identifying attributes of non-attending women to regular time intervals entails going beyond demographic differences to reveal complex interactions among personality attributes. Consistent with Champion’s Health Belief Model and Leventhal’s Common-Sense Model of self-regulation, it was the ‘perceived barriers’ and ‘cues to action’ constructs that emerged as the strongest predictors to describe the variance between the ADHERENT and NON-ADHERENT groups. Hence, women who attend at longer intervals may need to overcome barriers to seeking mammography and follow tailored cues to action in order to attend at recommended time intervals.

A plausible explanation for the disappearance of an effect of socio-demographic factors in our subgroup analyses on adherence in this study is that they represent ‘carriers’, as described by Lagerlund [65], of already established health-related behaviours. This is evidence in all our studies on first invitation to MBSP [13], re-attendance [14] and lifetime mammography use [63], where different socio-demographic and health status variables were non-significant predictors of uptake to mammography screening.

Literature suggests that having a breast condition or symptoms increase the use of mammography [66, 67] but this factor has not been found consistently in all studies [27, 68] and similarly, not in this study on timely mammography adherence. These results can indicate trust in the health care system and positive cancer experiences such as family members or close friends surviving cancer, but this issue needs further attention, preferable in qualitative research.

In all our data analyses, knowledge of the BS frequency was found to be significantly associated with MBSP attendance [13], re-attendance [14], lifetime screening [63] and likewise in this study on timely mammography adherence, showing that women who were unsure were less likely to attend for a mammogram at recommended intervals. Ritvo et al. [19] expands on such data, showing that it becomes more consequential with findings that the belief about recommended screening intervals predicts screening adherence in women with a family history of BC. Our findings are consistent with studies that examined the relationship in average risk women over 50 years where women who reported screening according to the respective national guidelines were significantly more likely to adhere than women who reported less frequent time intervals [19, 69, 70]. Our results underscore the significance of communicating and reiterating a screening interval recommendation to women such that they develop strong beliefs about the need to screen at that recommended time interval.

In some countries with dual public and private screening programmes, there is only partial understanding of adherence. Women who were active in opportunistic screening, for instance, are considered non-compliant to the screening programme [27]. Moreover, varying patterns of opportunistic screening exist (differing age groups, sometimes single view mammography) [71], including varying screening intervals such that women’s last mammography may have been longer than the recommended screening guidelines [9, 72].

The reasons why women chose to opt for private mammography rather than to the organized programme are not yet fully understood. This may be due to women seeking BS at a younger age (30–49) as a precautionary measure [20] and continue to sustain early detection practices in this way. Evidence from the U.S. National Health Interview Survey revealed that 29% of women aged 30–39 have undergone mammography [73] while data from the 2010 Behavioural Risk Factor Surveillance Study showed that 83% of women aged 40–49 have had BS [74]. Hence, public health strategies and wide media coverage directed at convincing older women to engage in BS may arouse a positive attitude among younger age groups towards early detection practices [75, 76] or may induce anxiety and fear of BC and mortality, motivating younger women, particular those aged 40–49, to engage in mammography screening [20].

The balance of benefits and harms remains to date a strongly debated topic in the field of population-based BS [77]. Although the usually considered benefits from BS include avoiding deaths from BC, achieving less invasive treatments and improving quality of life, there is growing concern that mammography may be overused (overscreening) [78], or screening may result in the detection by screening of BCs that would never have come to clinical attention (overdiagnosis) [79] and thus women receiving treatment for a slow growing or non-invasive cancer which would have unlikely caused any problems if left untreated [80]. Given the lack of reliable evidence, an independent expert panel estimated that around 1 in 4 women (or 4000 out of around 15,500 women) are overdiagnosed in the UK [81]. This is coupled with the side-effects and anxiety that anyone having cancer treatment goes through. Moreover, experts estimate that for every 10,000 women who have regular three-year screening between 47 and 73 years in the UK, there will be between 3 to 6 extra BCs caused by radiation [81]. Notwithstanding, the recent IARC Working Group [79] found sufficient evidence of a reduction in BC mortality through mammography screening in women aged 50–74 years, to the extent that the benefits substantially outweigh the risk of radiation-induced cancer and of overdiagnosis. Moreover, the survival rate of patients with early-detected BC through routine screening is approximately double that from detected cancers through other methods [81]. Unlike consistent mortality reductions reported through organised screening programmes [82], there is yet no direct evidence to support the effectiveness of opportunistic screening [83, 84].

Our results suggest that those attending an organised screening programme such as, the MBSP, are more likely to adhere to recommended time intervals when compared with those attending the private sector for screening. In order to reach greater adherence to recommended time intervals, women should receive further information on the recommended screening frequency and the benefits of being part of an organised programme [31, 85]. For example, in Malta, private screening does not have the same quality controls as the MBSP such as higher quality of mammographic interpretation through special training of the readers and mammographers, double-reading and consensus reads [71]. For instance, a mammogram in organised screening is read by two radiologists, who interpret at least 5000 screening mammograms a year. This is the recommended volume set by the European Guidelines for Quality Assurance in Breast Cancer Screening [30, 82, 84–86] and the desirable individual level of experience set in the United Kingdom [87]. Moreover, based on a similar screening context as our local system, other studies raise awareness that organized screening leads to inequality reductions, higher quality assurance, and more timely screens than opportunistic screening [27, 71, 83, 88].

While private screening remains unregulated, quality cannot be guaranteed which is why national screening programmes are recommended [71, 89]. Therefore, a key benefit of a national screening programme is that women can be individually monitored to ensure that they are conformant with screening guidelines and that they are adequately monitored in terms of robust quality assurance measures.

### Strengths and limitations

Although this study was limited to the Maltese setting, much of the developed world has organised breast screening programs and access to the same body of scientific evidence, and thus the findings are likely to be broadly applicable across these countries. However, we found no study that has similarly and simultaneously assessed sociodemographic and psychological variables as predictors of timely attendance and specifically attendance at organized or opportunistic screening. We also found none which utilised HBM and CSM as the theoretical framework. The major strength of this study is the rich dataset which allowed us to analyse diverse subgroups. However, this is not without possible response bias as a source of possible weakness. Since this study was cross-sectional, it precludes looking at cause-and-effect relationships over time. While a Chi-square test showed that women who attend private screening were less likely to be regulated in their attendance when compared to MBSP attendees, it cannot be ruled out that women attending private screening would be less likely to attend screening regularly even if they attended MBSP. This could be due to the characteristics of the women attending private screening. Although screening attendance was confirmed through screening records, our records of private mammography were self-reported and hence, subject to bias. Self-reported data could also have affected the observed difference between women attending private screening and the MBSP. Objective measurement would require data from private clinics, which was not possible to obtain, since no data records from private screening in Malta are nationally available to date. As a first step, it would be necessary to identify reliable and validated measures for regular mammography use that can be used simultaneously in government organised and private screening programmes. While limited studies to date have been of sufficient dimension to provide results on irregular attendance [16], qualitative studies would contribute towards understanding why health beliefs influence adherence.

## Conclusions

Our results suggest that attendance at an organised BS programme improves adherence to recommended time intervals when compared with those attending the private sector for screening. In order to reach greater adherence to recommended time intervals, women should be made more aware of the recommended screening frequency and on the benefits of being part of an organised programme. Women can be individually monitored through the national screening programme to ensure that they are conformant with screening guidelines. Screening programmes should target women’s health beliefs, in particular perceived barriers and cues to action, which have emerged as the most important factors to distinguish between adherent and non-adherent women to improve adherence to recommended time intervals. Further qualitative research is required to understand in more depth why women choose opportunistic screening over an organised programme.

## Additional file

Additional file 1: STROBE 2007 (v4) Statement—Checklist of items that should be included in reports of *cross-sectional studies*. (DOCX 22 kb)

## Abbreviations

B: Unstandardized coefficients
*BC*: Breast cancer
BS: Breast screening
CHBMS-MS: Champion’s Health Belief Model Scale for Mammography Screening
CI: Confidence interval
CSM: Common-sense model
DNA: Did not attend
GP: General practitioner
*HBM*: Health belief model
IPQ: Illness perception questionnaire
IPQ-R: Revised illness perception questionnaire
M: mean score
MBSP: Maltese Breast Screening Programme
OR: Odds ratio
SD: Standard deviation
SE: Standard error
WHO: World Health Organization
